# Validity, responsiveness and the minimal clinically important difference for the de Morton Mobility Index (DEMMI) in an older acute medical population

**DOI:** 10.1186/1471-2318-10-72

**Published:** 2010-09-30

**Authors:** Natalie A de Morton, Megan Davidson, Jennifer L Keating

**Affiliations:** 1 School of Primary Health Care, Faculty of Medicine, Nursing and Health Sciences, Monash University - Peninsula Campus, PO Box 527, Frankston, Victoria, 3199, Australia; 2 The Northern Clinical Research Center, Northern Health, 185 Cooper St, Epping, Victoria, 3076, Australia; 3 School of Physiotherapy, Division of Allied Health, Faculty of Health Sciences, La Trobe University, Victoria, 3086, Australia

## Abstract

**Background:**

The de Morton Mobility Index (DEMMI) is a new mobility instrument that overcomes the limitations of existing instruments. It is the first mobility instrument that accurately measures the mobility of all older adults. The aim of this study was to provide a detailed report of investigations of the validity, responsiveness to change and minimal clinically important difference (MCID) of the DEMMI during its development in an older acute medical population.

**Methods:**

This study was conducted using a head to head comparison design in two independent samples of older acute medical patients (development sample, *n *= 86; validation sample, *n *= 106). Consecutive patients (≥ 65 years) were assessed using the DEMMI, Barthel Index (BI) and Hierarchical Assessment of Balance and Mobility (HABAM) within 48 hours of hospital admission and discharge. Convergent and discriminant validity were investigated using Spearman's rho and known groups validity was investigated using a independent t test to compare DEMMI scores for patients who were discharged to home compared to inpatient rehabilitation. Criterion and distribution based methods were employed for estimating instrument responsiveness to change and the MCID.

**Results:**

Significant moderate to high correlations were identified between DEMMI and BI scores (r = 0.76 and r = 0.68) and DEMMI and HABAM scores (r = 0.91 and r = 0.92) in both samples. In both samples, DEMMI scores for patients who were discharged to home were significantly higher than for patients discharged to inpatient rehabilitation and provided evidence of known groups validity. Patients who were discharged to inpatient rehabilitation (*n *= 8) had a mean DEMMI score of 50.75 (sd = 11.29) at acute hospital discharge compared to patients who were discharged to home (*n *= 70) with a mean DEMMI score of 62.14 (sd = 18.41). MCID estimates were similar across samples using distribution and criterion based methods. The MCID for the DEMMI was 10 points on the 100 point interval scale. The DEMMI was significantly more responsive to change than the BI using criterion and distribution based methods in the validation sample.

**Conclusion:**

This study has validated the DEMMI in two independent samples of older acute medical patients. Estimates of its responsiveness and MCID have also been established. This study confirms that the DEMMI overcomes the limitations of the BI and HABAM and provides an advanced method for objectively assessing mobility for older acute medical patients.

## Background

Despite the many health benefits of maintaining physical independence in older age, two systematic reviews [1,2] identified no mobility instrument that could accurately measure and monitor changes in mobility for older patients from the acute hospital setting back to full health in the community. These findings led to the development and validation of the de Morton Mobility Index (DEMMI) in the acute hospital setting. The DEMMI was designed to overcome the limitations of existing instruments and was developed based on the Rasch model [3].

Clinimetrically sound instruments are essential to assist healthcare professionals to accurately measure and monitor changes in patient health, to assess the efficacy of interventions and to facilitate goal setting for therapeutic intervention. Instrument measurement properties, such as validity, responsiveness to change and the minimal clinically important difference (MCID) are required for confidence in interpretation of measurements. During instrument development, it is also important to establish that measurement properties are acceptable and that measurement stability is confirmed in an independent validation sample.

Validity refers to the extent to which inferences can be made from measurements based on the ability of the instrument to measure the construct of interest. There are many different types of validity that can be established using a variety of methods. Traditionally, validity has been established by content, criterion and construct validity [4]. However, there has been a proliferation of arguments regarding different methods for validating the extent to which measurements capture relevant information.

Face validity relates to whether the items appear, on the surface, to be measuring the construct of interest. Content validity refers to extent to which the test content is relevant to and representative of the construct of interest. Both face and content validity are usually obtained through the consensus of experts in the field.

Construct validity is now generally considered to be an overarching concept that encompasses criterion, convergent, discriminant and known groups validity [4]. Evidence for construct validity is obtained by showing that a test is related to performance on other theoretically related tests.

Criterion validity refers to the correlation of a scale with a direct measure of the construct of interest. In the absence of a 'gold standard' measure for the construct of interest (as is the case for the construct of mobility), convergent, discriminant and known groups validity can be investigated to obtain evidence for construct validity. Evidence for convergent validity is provided if test scores correlate with other measures of the same construct and evidence of discriminant validity is established if scale scores do not correlate with measures of unrelated constructs. Evidence of known groups validity is obtained if groups who are known to differ on the construct of interest score differently on the test. Predictive validity can also provide evidence of construct validity by indicating scores on one variable predicting outcome on another variable.

Responsiveness refers to the extent to which a measurement tool can detect important change. The importance of the concept of 'responsiveness' or 'sensitivity to change' is debated: some argue that responsiveness is a less important measurement requirement than reliability and validity, others contend that responsiveness provides evidence of validity and yet others propose that responsiveness is the most important quality of measurements [5].

In a systematic review of the responsiveness of health related quality of life instruments, Terwee et al.[5] identified 31 responsiveness indices. Most indices could be classified as either evaluating change over time (distribution based methods) or clinically important change over time (criterion based methods). They concluded that comparisons of the responsiveness of different instruments can lead to different conclusions depending on the index selected. For the purposes of evaluating existing mobility instruments in this study both distribution and criterion based methods were planned.

The MCID was defined by Jaeschke, Singer and Guyatt [6] as "the smallest difference in score in the domain of interest which patients perceive as beneficial......." The MCID provides clinicians with the change in scores that patients perceive to represent an important amount of change.

We have argued [7] that the reliability of an instrument is an important measurement property as it provides clinicians and researchers with an estimate of measurement error associated with instrument application. The minimal change that exceeds error in 90% of cases (MDC_90_) has been repeatedly verified for the DEMMI as 9/100 points on the Rasch converted interval scale [3,7].

The aim of this study was to investigate the validity, responsiveness to change and the MCID for Rasch converted DEMMI scores obtained in two independent samples. In the DEMMI development sample, a subset of 17 mobility items was selected from a larger pool of 42 tested items to construct the DEMMI. These items fitted the Rasch model and therefore provided evidence that a unidimensional mobility instrument had been achieved [3]. In the DEMMI validation sample, these 17 items were tested and 2 items were subsequently removed. It is theoretically plausible that items might operate differently when tested within a larger set of items (i.e. 17 items within a pool of 42 tested items) due to learning or fatigue effects compared to the final set of 15 item DEMMI. Nevertheless it was expected that the DEMMI development study would provide data to estimate the measurement properties required to interpret scores obtained using the DEMMI before proceeding to the next stage of instrument development. Clinimetric data obtained from the development sample also provided estimates for comparison with estimates obtained in the independent validation sample.

As part of validation against a reference standard, comparison with other instruments that have been used to measure mobility was planned. In order to compare the performance of different instruments that propose to measure the same underlying construct, a head-to-head comparison was the preferred design. Data obtained for different instruments from different samples could provide misleading evidence of relative utility as factors such as sample variance affect estimations. These confounders can be countered when comparisons of measurement properties are made using data collected at the same point in time from the same participants. We therefore included in this study a head-to-head comparison of the DEMMI with the Barthel Index [8] and Hierarchical Assessment of Balance and Mobility (HABAM)[9] in two independent samples of older acute medical patients.

## Methods

Data were collected as previously reported by de Morton, Davidson and Keating (2008) [3] in two independent samples of older acute medical patients. Participants were acute medical patients admitted to The Northern Hospital, Australia who were aged 65 years and older. Written and informed consent was obtained from each participant (either first or third party consent) within 48 hours of admission. In the DEMMI development sample (n = 89), 42 mobility items were tested (de Morton, Davidson, Keating. The development of the de Morton Mobility Index (DEMMI) in an older acute medical population: item reduction using the Rasch model (PART 1). Submitted) and in the DEMMI validation sample (n = 106), 17 DEMMI items were tested on each participant within 48 hour of admission and every 48 hours until discharge from the acute hospital setting (de Morton, Davidson, Keating. The development of the de Morton Mobility Index (DEMMI) in an independent sample of older acute medical patients: refinement and validation using the Rasch model (PART 2). Submitted). This study was approved by the Northern Health and Monash University Human Ethics Committees.

Activity limitation measures that were selected for a head-to-head comparison with the DEMMI mobility items were the Barthel Index (BI)[8] and the HABAM [9]. The BI is one of the most widely used functional outcome measures and the HABAM was identified in a systematic review [1] to have the most desirable clinimetric properties of existing mobility outcome measures for an older acute general medical population. The modified BI[8] was employed in this study as this was the version already in use in a concurrent randomised controlled trial that included the same patient population at The Northern Hospital.

The modified BI is an ordinal scale that provides a total score between 0 and 100 where higher scores indicate greater independence in activities of daily living [8]. The HABAM is an interval level mobility instrument that provides a score between 0 and 26 [9]. Higher scores indicate increasing levels of independent patient mobility. In addition, the MMSE was conducted at each assessment. The MMSE is reported to be a valid and reliable measure of patient cognition [10]. It provides a score between 0 and 30 points where increasing scores indicate higher cognitive ability.

At each assessment, a research assistant administered the BI and the MMSE. The BI was administered by scoring patient response when asked each item on the scale. If the patient was confused or disorientated to time, place or person, the BI was administered by interviewing the ward nurse caring for the patient or a family member. As close as possible after the completion of the BI and MMSE, the patient was asked to complete the physical performance mobility items by the research physiotherapist, who was blinded to the BI score. The BI was always administered first so that BI self report scores were not influenced by patient performance on the mobility items. There was no persuasive reason why self report BI scores would influence physical performance on the mobility items. This method allowed a blinded head-to-head comparison of the DEMMI mobility items with the BI.

Only one item on the HABAM, *unlimited walking*, was not included in the mobility items for testing as it could not be objectively assessed. A patient was deemed to have successfully completed the *unlimited walking *item on the HABAM if they completed the *50 meter walk independently without a gait aid *item with ease. Since all of the other items contained in the HABAM were tested with the mobility items, the HABAM item scoring protocols were applied and a HABAM score was obtained post hoc after viewing the mobility items. A HABAM score was recorded for each mobility assessment by the research physiotherapist. This permitted a practical but non blinded head-to-head comparison of the HABAM with the DEMMI.

Concurrent with collection of measurements using the DEMMI, HABAM and BI, data were also gathered using a global rating of change (GRC) scale to provide a method for estimating criterion based responsiveness and the MCID. At each 48 hour assessment the patient and therapist completed the global change scale (5 point scale: much worse, bit worse, same, bit better, much better) comparing each patient's current mobility status to their mobility at admission assessment. An important limitation associated with this method of criterion based assessment is that responses can be influenced by many factors. Recall bias and cognitive impairment (chronic or acute) were deemed to be the factors most likely to confound GRC in this study. In addition, it was possible that patient's perceptions of the influence of their responses on decisions regarding readiness for discharge and discharge destination might also influence some patient reports of change in mobility status.

Significant cognitive impairment would also render some patients incapable of providing a valid GRC. To ensure that at least one GRC score was obtained for each patient who completed a 48 hour mobility assessment, therapist GRC was also recorded at each 48 hour assessment. This also provided data for comparing global change scores obtained by patients and therapists.

Many different GRC scales have been reported and vary considerably in the number of response options offered to raters. Streiner and Norman (1995)[4] argued that the minimum number of categories used by raters should be in the region of five to seven and that most people are unable to discriminate beyond seven categories. In the present study, response options were kept to the recommended minimum of five. This was done to maximise patient participation across the broad range of anticipated cognitive ability and with consideration of the significant proportion of people for whom English was not a primary language. A 5 point scale was adopted in the absence of arguments for an alternate method. It appeared to provide response options to discriminate between important categories of change in a simple and practical form.

### Data analysis

Rasch-converted interval level DEMMI scores from data gathered from older acute medical patients in the development (n = 86) and validation (n = 106) samples were used to investigate clinimetric properties of the DEMMI and to compare these properties to those of the BI and HABAM. Data analysis was performed using SPSS 12.0 (SPSS Inc., Chicago, USA) and Microsoft Excel 2002 (Microsoft Corporation, Redmond, USA).

In the DEMMI development sample, clinimetric properties were calculated from the 17 item DEMMI (i.e. the *jog *and *single leg balance with eyes closed *items were included)[3]. In the validation sample these were calculated for the 15 item DEMMI (i.e. the *jog *and *single leg balance with eyes closed *items were not included)[3].

### Validity

The following *a priori *hypotheses were tested to investigate convergent, discriminant and known groups validity for the DEMMI.

*A priori *hypotheses:

1. **Convergent validity: **DEMMI scores will have a significant and high correlation with BI and HABAM scores.

2. **Discriminant validity: **DEMMI scores will have low correlation with the MMSE, APACHE 11 severity of illness and Charlson co-morbidity scores.

3. **Known groups validity: **Patients who are discharged to home will have significantly higher DEMMI scores at hospital discharge than patients discharged to rehabilitation.

A Pearson's correlation coefficient or Spearman's rho and associated 95% confidence interval were calculated to investigate the convergent and discriminant validity of the DEMMI. To investigate the known groups validity, an independent t test was performed to compare mobility scores at hospital discharge for patients who were discharged to home compared to inpatient rehabilitation.

### Responsiveness

Measurement responsiveness was calculated using acute hospital admission and discharge scores on the DEMMI, HABAM and BI. Two responsiveness indices were selected *a priori*. A superior responsiveness index has not been reported in the literature and therefore indices were selected primarily due to their simplicity and known methods for calculating a 95% confidence interval. A distribution based index, the Effect Size Index (ESI)[11], and a criterion based index, Guyatt's Responsiveness Index (GRI)[12], were calculated for each activity limitation outcome measure. These indices were calculated using the following formulas:

$$\text{ESI}=\frac{\text{average}\;\text{change}}{\text{standard}\;\text{deviation}\;\text{of}\;\text{initial}\;\text{scores}}$$

$$\text{GRI}=\frac{\text{average}\;\text{change}\;\text{in}\;\text{change}\;\text{group}}{\text{standard}\;\text{deviation}\;\text{of}\;\text{change}\;\text{in}\;\text{stable}\;\text{group}}$$

For both the ESI and GRI, the 95% confidence interval was calculated by initially calculating the standard error of the estimate of the mean change in scores between admission and a subsequent test (change score sd/$\sqrt{\left(n-1\right)}$). These values were then adjusted for 95% confidence and inserted into the numerator of each formula to calculate the upper and lower limit of the 95% confidence interval for both indices.

GRI was calculated twice, once using patient GRC scores and again using therapist GRC scores. It has been argued that when patients rate their change as the 'same', 'bit better' or 'bit worse' it is unlikely that clinically important change has occurred [13,14]. Subsequently, patients in the categories of either 'bit better' or 'bit worse' were reclassified as 'unchanged' and also included in the reliability analysis [7]. Patient and therapist were not required to have absolute agreement about rating (i.e. 'bit worse', 'same' or 'bit better') and were classified as 'unchanged' if both opted for any one of those three responses. Decisions rules for identifying 'changed' and 'unchanged' patients for analyses were established *a priori*.

In this study, the responsiveness of the DEMMI was compared to the responsiveness of the BI and the HABAM using the ESI and GRI based on patient and therapist rating of change. Tryon (2001)[15] provides a useful method for adjusting for error in point estimates for multiple pairwise comparisons. Tryon established that the 95% confidence bands of two independent means can overlap yet remain significantly different at the 0.05 level. If the standard errors of two independent means are equal, Tryon reported non overlapping 84% confidence bands to indicate a significant difference between means. Using the methods described by Tryon [15], the width of inferential confidence bands are determined by t_crit _and a factor of E whilst maintaining an overall alpha level of 0.05 for multiple comparisons. T_crit _is determined by the number of pairwise comparisons and degrees of freedom (based on sample size). E is defined by Tryon as the "ratio of the standard error of the difference between groups to the sum of the standard errors of both groups." When data are presented using graphs that demonstrate group means and Tryon adjusted 95% confidence bands, statistical difference for any pairwise comparison can be inferred when confidence intervals do not overlap.

### Minimal clinically important difference

The MCID was calculated for the DEMMI, HABAM and BI using distribution and criterion based methods. The distribution based approach recommended by Norman et al. (2003)[16] was employed by using half the baseline standard deviation of admission scores as a best estimate of the MCID.

Clinically important change was considered to have occurred for patients who were rated as 'much better' at discharge assessment. Criterion based MCID estimates were obtained by calculating the average change in DEMMI scores for the 'changed group' between hospital admission and discharge. A criterion based MCID estimate was obtained using patient, therapist and when either therapist or patient reported they were 'much better'.

## Results

DEMMI, HABAM and BI assessments were completed at initial assessment for patients in the development (n = 86) and validation samples (n = 106). The characteristics of participants and the flow of participants in this study have been previously reported [3].

### Distribution of activity limitation scores at hospital admission

**Figure 1:**
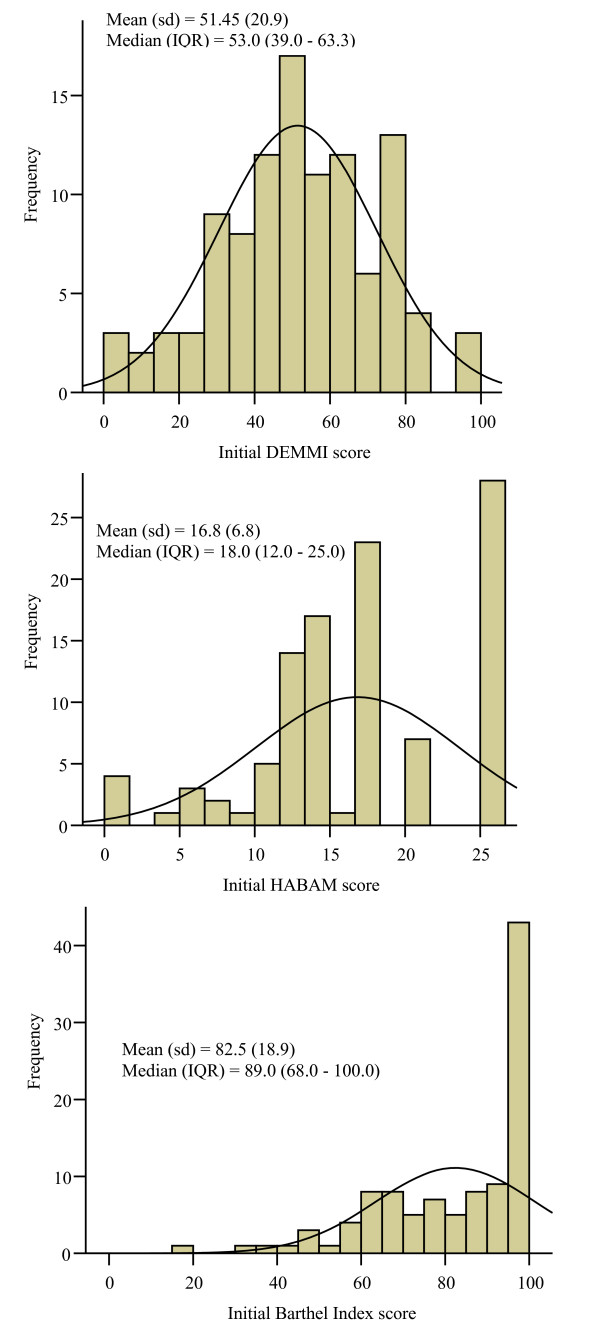
**a, b, c. Distribution of initial scores (DEMMI, HABAM and Barthel Index) in the validation study**.

The distribution of activity limitation scores at hospital admission were consistent across the two independent samples. Initial scores for the HABAM and BI were not normally distributed and had a ceiling effect. In contrast, initial DEMMI scores were normally distributed and did not display floor or ceiling effects in either sample (Figures 1a-c). In the validation sample, normal distribution of DEMMI scores was evidenced by similar mean and median scores, 69.8% of scores lying within one standard deviation (31 to 72) of the mean and skewness and kurtosis indices were -0.23 and 0.24 respectively. Only 3 patients (2.8%) scored above 90 and 5 patients (4.7%) scored less than 10 at their initial assessment. For the BI, 43% of patients scored 90 or higher and for the HABAM, 26% of patients scored either 25 or 26 at hospital admission. Table 1 shows the mean initial DEMMI scores for each major diagnostic category within the general medical patient population and indicates that the DEMMI has the scale width required to measure and monitor changes in mobility for each of these diagnostic groups.

**Table 1:** Initial DEMMI scores for each diagnostic group in the validation study.

Diagnostic group	*n*	Mean (sd)
Respiratory	37	55.95 (22.06)
Cardiovascular	21	59.10 (11.11)
Digestive	7	50.29 (18.45)
Endocrine	6	38.17 (21.97)
Genitourinary	6	43.67 (22.32)
Other	29	44.83 (22.39)

### Activity limitation change scores between hospital admission and discharge

Of the 106 patients included in the validation study, 67 patients had at least two assessments completed. Of these 67 patients, a therapist GRC score between hospital admission and discharge was obtained for 57 patients and a patient GRC score was obtained for 61 patients. The 6 patients who did not complete a GRC score were significantly older (p = 0.02) than patients who did complete a GRC score but were similar on all other baseline characteristics.

Table 2 shows patient and therapist GRC scores and accompanying change scores for the 15 item DEMMI, the HABAM and BI between acute hospital admission and discharge. Consistent with findings in the development sample, most patients were reported to improve in their mobility between acute hospital admission and discharge. No patients were reported to be 'much worse' by the therapist and there was only one report of 'much worse' by a patient. Two patients were reported by the therapist to be a 'bit worse' and three patients were rated by patients to be a 'bit worse.' Change scores between rating categories for patients reported to be 'bit worse', 'same' or 'bit better' (i.e. were classified as 'unchanged') were small. An independent t test identified no statistically significantly difference for change scores between patients reported to be the 'same' or 'bit better.' There were too few patients in the 'bit worse' category to provide meaningful point estimates for comparison.

**Table 2:** Global rating of change scores and DEMMI (0 - 100), HABAM (0 - 26) and BI (0 - 100) mean change scores (sd) between hospital admission and discharge in the validation study.

	Much worse	Bit worse	Same	Bit better	Much better
***Therapist***	*n *= 0	*n *= 2	*n *= 6	*n *= 26	*n *= 23

**DEMMI**	NA	-16.50 (3.54)	+3.67 (4.76)	+3.31 (7.19)	+14.00 (9.19)
**HABAM**	NA	0 (0)	0.43 (3.55)	+0.85 (2.98)	+4.70 (3.94)
**Barthel Index**	NA	-1*	-1.43 (15.04)	-0.15 (11.95)	+8.09 (11.00)

**Patient**	*n *= 1	*n *= 3	*n *= 7	*n *= 14	*n *= 35

**DEMMI**	-19	-1.67 (10.69)	+10.14 (11.60)	+6.64 (9.25)	+9.43 (10.56)
**HABAM**	0	-2.00 (3.46)	+2.43 (4.04)	+2.29 (4.50)	+3.11 (3.92)
**Barthel Index**	NA*	-9.33 (15.31)	+11.5 (12.60)*	-1.64 (11.87)	+5.89 (12.13)

NA = Not applicable

* one BI assessment not obtained

For patients classified as 'unchanged' based on patient report of change, scores were significantly higher at hospital discharge compared to hospital admission for the DEMMI and HABAM but not for the BI (Table 3). For patients classified as 'unchanged' by the therapist, scores for the DEMMI, HABAM and BI were not significantly different at hospital admission compared to discharge (Table 3).

**Table 3:** DEMMI, HABAM and BI scores for patients rated 'unchanged', 'same' and 'changed' in the validation study.

	*n*	Admission mean (sd)	Discharge mean (sd)	p value	*n*	Admission mean (sd)	Discharge mean (sd)	p value
**Patient rating of change**					**Therapist rating of change**			

***Unchanged ('bit worse', 'same' or 'bit better')***								

**DEMMI**	24	45.38 (18.59)	52.00 (19.77)	0.01*	34	52.29 (20.19)	54.50 (20.21)	0.12
**HABAM**	24	15.00 (6.58)	16.83 (6.66)	0.05*	34	17.09 (6.55)	17.76 (6.04)	0.20
**BI**	23	76.35 (18.17)	77.13 (25.05)	0.79	34	81.41 (21.05)	81.79 (22.07)	0.86

***Same***								

**DEMMI**	7	48.14 (24.33)	58.29 (29.55)	0.06	6	45.67 (24.37)	49.33 (26.42)	0.12
**HABAM**	7	15.29 (8.67)	17.86 (9.46)	0.14	6	15.67 (8.36)	15.83 (9.00)	0.92
**BI**	6	73.5 (16.21)	85.0 (24.23)	0.08	6	77.17 (19.14)	75.50 (29.57)	0.81

***Changed ('much better')***								

**DEMMI**	35	53.06 (18.31)	62.49 (15.56)	0.00*	23	48.22 (14.89)	62.22 (13.55)	0.00*
**HABAM**	35	16.83 (6.18)	19.94 (4.81)	0.00*	23	15.17 (5.75)	19.87 (4.87)	0.00*
**BI**	35	82.34 (22.42)	88.23 (15.13)	0.01*	23	81.35 (20.32)	89.43 (15.86)	0.00*

*p < 0.05

For patients classified as 'changed' by the patient and the therapist, DEMMI, HABAM and BI scores were significantly higher at hospital discharge compared to hospital admission (Table 3). However, a broad range of change scores were classified as both 'changed' and 'unchanged' by both patients and therapist.

### Validity

#### Convergent validity

**Figure 2:**
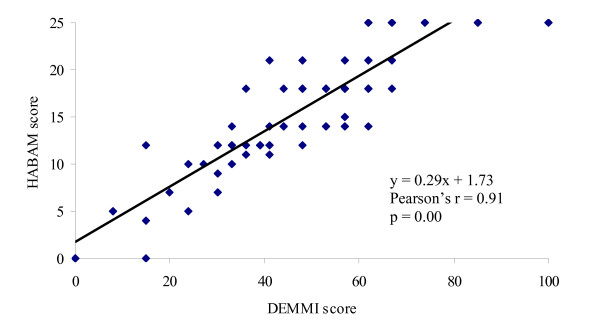
**Scatterplot of DEMMI and HABAM scores at initial assessment in the validation study**.

**Figure 3:**
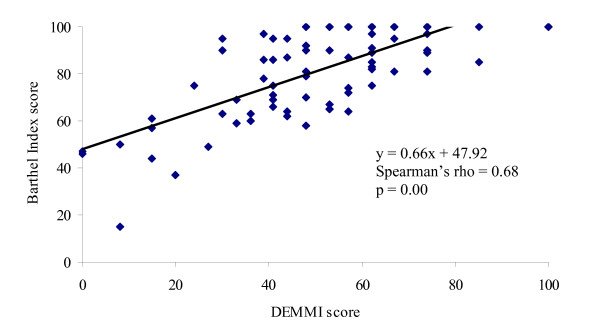
**Scatterplot of DEMMI and BI scores at initial assessment in the validation study**.

Evidence of convergent validity for the DEMMI was obtained in both samples by identifying a significant and high correlation with HABAM scores (*Development sample: *Pearson's r = 0.92, 95% CI 0.88 to 0.95, p = 0.00; *Validation sample: *Pearson's r = 0.91, 95% CI 0.87 to 0.94, p = 0.00, *n *= 106, Figure 2) and a moderate to high correlation with BI scores (*Development sample: *Spearman's rho = 0.76 95% CI 0.65 to 0.84, p = 0.00; *Validation sample: *Spearman's rho = 0.68 95% CI 0.56 to 0.77, p = 0.00, *n *= 105, Figure 3) at initial assessment. The relationship between these measures was obscured by the HABAM and BI ceiling effect.

#### Discriminant validity

Discriminant validity for the DEMMI was evidenced in both samples by a low correlation with measures of other constructs. Initial DEMMI scores had a significant but low correlation with the MMSE (Development sample: Spearman's rho = 0.36 95% CI 0.16 to 0.53, p = 0.00, *n *= 85; Validation sample: Spearman's rho = 0.24, 95% CI 0.05 to 0.41, p = 0.02, *n *= 103), and low and non significant correlations with APACHE 11 severity of illness scores (*Development sample: *Spearman's rho = -0.11 95% CI -0.32 to 0.11, p = 0.34, *n *= 83; *Validation sample: *Spearman's rho = 0.07, 95% CI -0.12 to 0.26, p = 0.49, *n *= 105) and Charlson co-morbidity index scores (*Development sample: *Spearman's rho = -0.19 95% CI -0.39 to 0.03, p = 0.08, *n *= 84; *Validation sample: *Spearman's rho = -0.04, 95% CI -0.23 to 0.15, p = 0.68, *n *= 105).

#### Known groups validity

In both samples, an independent t test showed that patients who were discharged to inpatient rehabilitation had significantly lower DEMMI scores at acute hospital discharge than those discharged to home, providing evidence of known groups validity for the DEMMI. In the development sample, patients discharged to inpatient rehabilitation (*n *= 11) had a mean DEMMI score of 39.55 (sd = 9.41, 95% CI 33.72 to 45.38) and patients discharged to home (*n *= 62) had a mean DEMMI score of 59.61 (sd = 13.22, 95% CI 56.30 to 62.93) (p = 0.00). In the validation sample, patients who were discharged to inpatient rehabilitation (*n *= 8) had a mean DEMMI score of 50.75 (sd = 11.29, 95% CI 42.39 to 59.11) at discharge compared to patients who were discharged to home (*n *= 70) with a mean DEMMI score of 62.14 (sd = 18.41, 95% CI 57.80 to 66.49). An independent t test of discharge DEMMI assessment scores for these two groups showed these scores to be significantly different (p = 0.03).

#### Responsiveness to change

**Figure 4:**
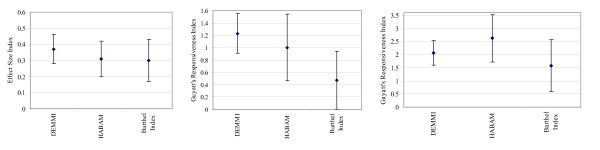
**a, b & c. Development sample**: a) Effect Size Index with 87.3% confidence intervals. b) Guyatt's Responsiveness Index based on patient rating of change with 88.3% confidence intervals. c) Guyatt's Responsiveness Index based on therapist rating of change with 89.5% confidence intervals.

In the development sample, there was no significant difference identified between the responsiveness of DEMMI and HABAM measurements or DEMMI and BI measurements using the ESI or GRI based on patient or therapist report of change. This is shown by overlapping Tryon adjusted inferential confidence intervals in Figures 4a-c.

**Figure 5:**
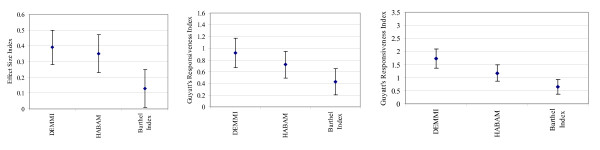
**a, b & c. Validation sample**: a) Effect Size Index with 83.7% confidence intervals. b) Guyatt's Responsiveness Index based on patient rating of change for with 84.4% confidence intervals. c) Guyatt's Responsiveness Index based on therapist rating of change with 84.9% confidence intervals.

However, in the validation sample, using the ESI and GRI based on patient report of change and GRI based on therapist report of change, DEMMI measurements were significantly more responsive to change than the BI but not the HABAM (Figures 5a-c). For the ESI, GRI based on patient report of change and GRI based on therapist report of change, t_crit _was calculated to be 2.28, 2.34 and 2.40 respectively.

### Minimal clinically important difference

#### Criterion based MCID

The MCID was estimated by calculating the average change in DEMMI score for the 'changed' group between hospital admission and discharge. Table 4 shows similar criterion based MCID estimates obtained using patient, therapist, and patient or therapist ratings of change from both samples. Patient report led to estimates of clinically important change in mobility that were smaller than those based on therapist report in both samples.

**Table 4:** Criterion and distribution based MCID estimates for the DEMMI

	Criterion method			Distribution method
	**Patient** **(*n *= 23)**	**Therapist** **(*n *= 21)**	**Patient or therapist** **(*n *= 31)**	

**Development sample MCID** **(95% CI)**	7.78 (5.28 to 10.28)	10.43 (7.74 to 13.11)	8.45 (6.09 to 10.81)	8.00

	**Patient** **(*n *= 35)**	**Therapist** **(*n *= 23)**	**Patient or therapist** **(*n *= 42)**	

**Validation sample** **MCID (95% CI)**	9.43 (5.92 to 12.92)	14.00 (10.24 to 17.75)	9.71 (6.66 to 12.72)	10.46

#### Distribution based MCID

Using Norman et al.'s [16] distribution based method, the MCID was calculated to be 8.00 and 10.46 points for the DEMMI in the development and validation samples respectively. The MCID for the HABAM and BI were also obtained using this distribution based method and were calculated to be 3.4 out of 26 and 11.4 out of 100 points respectively in the development sample. In the validation sample, the MCID for the HABAM and BI were also obtained using this method and were calculated to be 3.39 and 9.43 points respectively.

## Discussion

In this study the measurement properties of the DEMMI have been validated in two independent samples of older acute medical patients. In a repeated head-to-head comparison with the HABAM and BI, this study also confirmed the superior measurement properties of the DEMMI and provides further evidence that the DEMMI offers researchers and clinicians a superior method for measuring and monitoring changes in mobility for older acute medical patients.

The DEMMI was confirmed in the validation sample to be a broad scale with few patients scoring within the MDC_90 _(9 points) of the scale extremes. The DEMMI did not have a floor or ceiling effect and had the scale width required to measure improvement and deterioration in mobility for each of the most common medical diagnostic subgroups within an older general medical population. In contrast, a ceiling effect was confirmed for both the BI and HABAM with a large proportion of patients scoring at the upper scale extreme at their initial assessment. Since the DEMMI has a broad scale width and is safe and practical for application in older acute medical patients, the DEMMI also has the potential to measure changes in mobility after acute hospital discharge (e.g. in the subacute hospital or community settings).

Similar evidence of scale convergent, discriminant and known groups validity was observed in the development and validation samples. The DEMMI had high correlation with measures of related constructs (the HABAM and BI), low correlation with measures of other constructs (MMSE, APACHE 11 and Charlson co-morbidity Index) and patients who were discharged to home had significantly higher DEMMI scores than patients who were discharged to inpatient rehabilitation.

In the development sample, there were no significant differences in the responsiveness of DEMMI compared to HABAM or BI measurements. The DEMMI had the highest responsiveness point estimate using the ESI and GRI based on patient ratings of change. However, the HABAM had the highest responsiveness index using GRI based on therapist rating of change scores. It was observed that the denominator in calculations of GRI for the HABAM (based on therapist rating of change scores) was much smaller than for the DEMMI and thus a larger responsiveness index was obtained for the HABAM. Closer examination of the raw data revealed that due to the HABAM ceiling effect, the standard deviation of initial scores for the unchanged group was relatively small. This indicates that for an instrument with a known ceiling effect such as the HABAM (that is therefore not responsive to change at the upper end of its scale) an artificially inflated criterion based responsiveness index can result from inadequate scale width.

Although the responsiveness of the BI and HABAM was not a comparison of interest in this study, MacKnight and Rockwood (1995)[17] reported the HABAM to be more responsive to change than the BI in an older acute medical population. Compared to estimates in this study, MacKnight and Rockwood reported a higher ESI of 0.51 for the HABAM and 0.35 for the BI. However, distribution based methods of calculating responsiveness to change are dependent on the magnitude of change that a group undergoes between assessments and is therefore population dependent. The patient population in this study was, on average, more independent in their activities of daily living and mobility at hospital admission than patients in the study reported by MacKnight and Rockwood. This may account for differences between study results. In addition, only 28 patients were included in the study reported by MacKnight and Rockwood and their conclusions appear to be based on the ranking of ESI point estimates, as the 95% confidence intervals surrounding these indices were not provided in the published report. These authors also reported the HABAM to be three times more responsive to change than the BI using the relative efficiency index. However, the relative efficiency was not calculated in this study as there are no established methods for calculating the 95% confidence interval for this responsiveness index.

In contrast to the development sample in this study, responsiveness indices in the validation sample identified the DEMMI to be significantly more responsive to change than the BI using the ESI and GRI (based on patient and therapist report of change). No significant difference was identified between the responsiveness of the DEMMI and the HABAM using either the ESI or GRI. Although responsiveness indices were calculated for the 17 item instrument in the development sample and for the 15 item instrument in the validation sample, these results highlight the sample dependency of responsiveness indices. Different results can be obtained in different samples as the DEMMI was identified to be significantly more responsive to change than the BI in the validation sample but not in the instrument development sample.

For outcome measure application, it is optimal to provide one MCID index rather than different indices obtained using different methods. After completing this study, an MCID estimate of 10 points was added to the final format of the DEMMI assessment form. This indicates that a change score of 10 points or more is required to represent a clinically important change in patient mobility.

The MCID estimates obtained in the final validation study were not significantly different to the estimates calculated from the instrument development sample. Consistency of these measurement properties across independent study samples also provides clinicians and researchers with further confidence in the interpretation and clinical application of DEMMI scores.

The MCID for the DEMMI, HABAM and BI represent 10.0%, 13.1% and 11.4% of the instrument scale widths respectively. For the DEMMI, only 3 patients (2.8%) scored above 90 and 5 patients (4.7%) scored less than 10 at their initial assessment in the validation sample and therefore the DEMMI provided the scale width required to adequately measure clinically important change for all older acute medical patients included in this study. In contrast, due to the HABAM and BI ceiling effect, these instruments were unable to measure clinically important change at the upper end of these scales for a large proportion of older medical patients included in this study.

This study had limitations. For the known groups validity analyses there were only 11 patients in the development sample and 8 patients in the validation sample who were discharged to inpatient rehabilitation. In addition, the relatively small sample sizes for the development (*n *= 86) and validation (*n *= 106) samples may also explain some of the differences that were identified between samples in this study.

## Conclusion

This study confirmed that the DEMMI is a valid mobility instrument with consistent MCID estimates across independent samples. These results indicate the validity of clinical and research application of the DEMMI in an older acute medical population. Estimates of responsiveness have also been reported. In a repeated head-to-head comparison with the HABAM and BI, this study has confirmed that the DEMMI overcomes the limitations of these two instruments and provides an advanced method for objectively assessing and monitoring changes in mobility for older acute medical patients.

## Competing interests

The authors declare that they have no competing interests.

## Authors' contributions

NdM was responsible for the conception, design, analysis of data and preparation of the manuscript under the guidance of co-authors as this work constitutes a component of Nd's doctoral studies with co-authors as doctoral supervisors (MD and JK). All authors approved the final manuscript.

## Pre-publication history

The pre-publication history for this paper can be accessed here:

http://www.biomedcentral.com/1471-2318/10/72/prepub

## References

[B1] de Morton N Berlowitz D Keating J A systematic review of mobility instruments and their measurement properties for older acute medical patients BMC Health and Quality of Life Outcomes 2008 6 10.1186/1477-7525-6-44 PMC2430553 18533045

[B2] Davenport S Paynter S de Morton N What instruments have been used to assess the mobility of community dwelling older adults? Physical Therapy Reviews 2008 13 345 354

[B3] de Morton N Davidson M Keating J The de Morton Mobility Index (DEMMI): an essential index for an ageing world BMC Health and Quality of Life Outcomes 2008 6 10.1186/1477-7525-6-63 PMC2551589 18713451

[B4] Streiner DL Norman GR Health Measurement Scales. A practical guide to their development and use 1995 Second New York: Oxford University Press

[B5] Terwee CB Dekker FW Wiersinga WM Prummel MF Bossuyt PMM On assessing responsiveness of health-related quality of life instruments:guidelines for instrument evaluation Quality of Life Research 2003 12 349 362 10.1023/A:1023499322593 12797708

[B6] Jaeschke R Singer J Guyatt GH Measurement of health status. Ascertaining the minimally clinically important difference Controlled Clinical Trials 1989 10 407 415 10.1016/0197-2456(89)90005-6 2691207

[B7] de Morton N Davidson M Keating J Reliability of the de Morton Mobility Index (DEMMI) in an older acute medical population Physiotherapy Research International in press 10.1002/pri.493 21043046

[B8] Shah S Vanclay F Cooper B Improving the sensitivity of the Barthel Index for stroke rehabilitation Journal of Clinical Epidemiology 1989 42 703 709 10.1016/0895-4356(89)90065-6 2760661

[B9] MacKnight C Rockwood K Rasch analysis of the hierarchical assessment of balance and mobility (HABAM) Journal of Clinical Epidemiology 2000 53 1242 1247 10.1016/S0895-4356(00)00255-9 11146271

[B10] Folstein MF Folstein SE McHugh PR "Mini-Mental State." A practical method for grading the cognitive state of patients for the clinician Journal of Psychiatric Reserve 1975 12 189 198 10.1016/0022-3956(75)90026-6 1202204

[B11] Kazis LE Anderson JJ Meenan RF Effect sizes for interpreting changes in health status Medical Care 1989 27 S178 189 10.1097/00005650-198903001-00015 2646488

[B12] Guyatt GH Walter S Norman G Measuring change over time: assessing the usefulness of evaluative instruments Journal of Chronic Disease 1987 40 171 178 10.1016/0021-9681(87)90069-5 3818871

[B13] Davidson M Keating JL A comparison of five low back disability questionnaires: Reliability and responsiveness Physical Therapy 2002 82 8 24 11784274 10.1093/ptj/82.1.8

[B14] Stratford PW Finch E Solomon P Binkley J Gill C Moreland J Using the Roland-Morris Questionnaire to make decisions about individual patients Physiotherapy Canada 1996 48 107 110

[B15] Tryon W Evaluating ststistical difference, equivalence, and indeterminacy using inferential confidence intervals: an integrated alternative method of conducting null hypothesis statistical tests Psychological Methods 2001 6 371 386 10.1037/1082-989X.6.4.371 11778678

[B16] Norman GR Sloan JA Wyrwich KW Interpretation of changes on health related quality of life. The remarkable universality of half a standard deviation Medical Care 2003 41 582 592 10.1097/00005650-200305000-00004 12719681

[B17] MacKnight C Rockwood K A Hierarchical Assessment of Balance and Mobility Age and Ageing 1995 24 126 130 10.1093/ageing/24.2.126 7793334

